# A hierarchy of environmental covariates control the global biogeography of soil bacterial richness

**DOI:** 10.1038/s41598-019-48571-w

**Published:** 2019-08-20

**Authors:** Samuel Bickel, Xi Chen, Andreas Papritz, Dani Or

**Affiliations:** Soil, Terrestrial and Environmental Physics (STEP), Institute of Biogeochemistry and Pollutant dynamics (IBP), Swiss Federal Institute of Technology (ETH), 8092 Zürich, Switzerland

**Keywords:** Soil microbiology, Microbial ecology, Biogeography, Biodiversity

## Abstract

Soil bacterial communities are central to ecosystem functioning and services, yet spatial variations in their composition and diversity across biomes and climatic regions remain largely unknown. We employ multivariate general additive modeling of recent global soil bacterial datasets to elucidate dependencies of bacterial richness on key soil and climatic attributes. Although results support the well-known association between bacterial richness and soil pH, a hierarchy of novel covariates offers surprising new insights. Defining climatic soil water content explains both, the extent and connectivity of aqueous micro-habitats for bacterial diversity and soil pH, thus providing a better causal attribution. Results show that globally rare and abundant soil bacterial phylotypes exhibit different levels of dependency on environmental attributes. Surprisingly, the strong sensitivity of rare bacteria to certain environmental conditions improves their predictability relative to more abundant phylotypes that are often indifferent to variations in environmental drivers.

## Introduction

Delineating biogeographical patterns of soil bacterial richness could offer insights into potential links between natural bacterial community traits and belowground ecological functioning^1^. Various external drivers, land use and biome characteristics shape the soil bacterial community composition and structure. Spatial mapping of soil bacterial richness remains a challenge due to the high number of bacterial phylotypes and the sparse global coverage of available samples^2–4^ that originate from only few biomes. The vast number of possibilities for community assembly across environments with high intrinsic heterogeneity limit inference of globally representative biogeographical patterns from small-scale measurements^3,5^. The establishment of reliable global maps of bacterial biogeography hinge on inclusion of ample sampling locations and tackle the hurdles of uneven sample sizes and primer biases in meta-analyses^6^. To overcome these limitations towards development of unbiased estimates of global bacterial richness patterns, comprehensive and well-harmonized data sets are required. Additionally, the primary drivers for soil bacterial richness are often obscured by large uncertainty in measurements and by sensitivity of species richness to methodology and sampling protocol^2^. Identifying drivers of bacterial richness is particularly error-prone due to the metrics sensitivity to the detection of rare and low abundant species; thereby challenges data analysis and interpretation. One of the most common predictor (covariate) of soil bacterial diversity is the soil pH^7–10^. For near neutral soil pH, bacterial diversity peaks and then drops for acidic and basic soils^7^. Some have argued that such a pattern reflects increased abundance of specialist species in such environments or, alternatively, that pH is merely a proxy for other environmental factors^7^. Along with soil pH, many other environmental characteristics, such as mean annual precipitation and mean annual temperature are expected to affect soil microbial life, yet their effects are difficult to assess independently as they are often interlinked and only partly exhibited at scales relevant to soil bacterial habitats^11,12^. Soil hydration status has emerged as a primary factor affecting soil bacterial habitats^13,14^, as supported by empirical observation^15–18^. The wetness of a soil affects the connectivity of the aqueous bacterial habitats^19^, thereby modifying interactions and the motility of bacterial cells that in turn affect community composition and diversity. Yet few attempts have been made to statistically test the dependency of bacterial diversity on climatic soil moisture conditions at the global scale.

Three recently published datasets of soil bacterial community composition^17,20,21^ combined with a consistent set of covariates (Supplementary Table S1) permit the (i) systematic consideration of composite soil and climate variables that could reflect salient conditions of soil bacterial habitats, and (ii) enable a process-based understanding of the hierarchy in environmental factors that control soil bacterial richness. In this study, we (iii) analyze biogeographic trends to statistically test the explanatory power of composite variables, specifically climatic water content, with respect to soil bacterial richness and (iv) predict global biogeographic trends using general additive models (GAM) and tree-based methods.

## Results and Discussion

**Figure 1:**
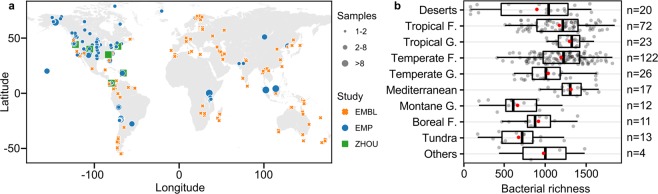
Distribution of sites and representative samples obtained from three recent studies (EMBL^17^, EMP^21^, ZHOU^20^) used in this meta-analysis. (**a**) Geographical locations of sites (n = 320, by continent: AF = 27, AS = 42, AU = 30, EU = 55, NA = 104, SA = 62). Size of the points represents the number of samples that were aggregated within 0.1° × 0.1° cells. Colors orange, blue and green represent the three studies EMBL, EMP and ZHOU respectively. (**b**) Bacterial richness grouped by biomes (F. forest, G. grassland). Site values are shown in grey, while the red points represent mean values. Boxes show the inter quartile range (median as solid line) with bars indicating central 95%-range of values.

Merging the geo-referenced 16S rRNA sequence data resulted in 844 valid soil samples, of which, 320 representative sampling sites were obtained after sample aggregation (Fig. 1a). Only bacterial diversity was analyzed, as the use of 16S rRNA sequences precludes the investigation of fungal diversity in the current study. Despite covering all 14 classified biomes of the world^22^, sampling was not even, and some biomes and continents were under- or overrepresented (e.g., deserts contribute to about 18.9% of the terrestrial surface, yet only 6.3% of samples originated from these environments). From a total of 256,620 amplicon sequence variants (ASV) detected, we removed Archaea and unassigned sequences (at kingdom level, 1.55%) leaving 98.45% of bacterial ASVs. For ease of communication, we refer to the designated bacterial ASVs as “species” throughout the text. The widest range of species richness was observed in deserts (Fig. 1b) and could be attributed to the wide span of variations in environmental conditions in such biomes^23^. The relatively low richness in montane grassland and tundra could be indicative of a non-monotonic relation between moisture availability and soil bacterial richness. Boreal forests (*n* = 11) exhibited lower richness compared to tropical (*n* = 23) and temperate forests (*n* = 122; *p* = 0.0311 and *p* = 0.0063, respectively, Wilcoxon rank sum test). This latitudinal shift in species richness^17,20^ suggests that temperature plays an important role in regulating bacterial richness. However, consideration of temperature alone provides no distinction between the richness observed in tropical and temperate forests (*p* = 0.6575, Wilcoxon rank sum test), suggesting more complex interactions and mechanisms.

### Univariate analysis of bacterial richness

**Figure 2:**
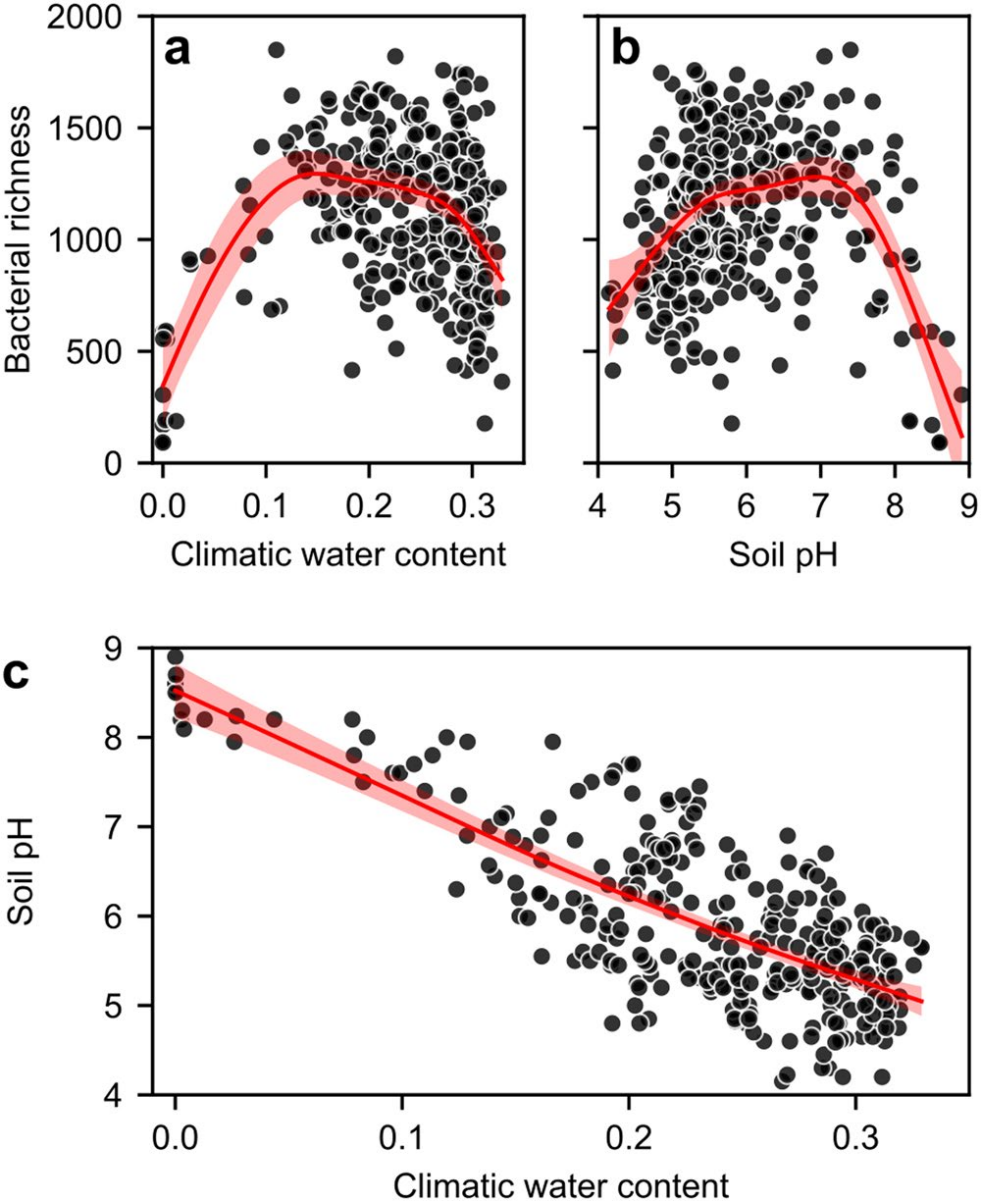
Univariate general additive model (GAM) of soil bacterial richness. (**a**) Relation between climatic water content and bacterial richness. Bacterial richness peaks in soils with intermediate climatic water contents (0.15–0.2) and drops in dry and wet soils (R^2^ = 27.7%, RMSE = 298.1, AIC = 4557.5, EDF = 4.7). (**b**) Commonly observed trend of bacterial richness peaking at near neutral conditions (pH 7) and showing distinct drops in acidic and basic soils (R^2^ = 23.8%, RMSE = 306.0, AIC = 4574.0, EDF = 5.1). (**c**) A strong linear association (adjusted R^2^ = 60.8%, deviance explained 61.1%) is observed between climatic water contents and soil pH pointing to possible confounding effects of these covariates on bacterial richness. Shaded areas correspond to standard errors (n = 320).

We first evaluate trends of species richness considering climate and soil properties within univariate general additive modeling. Selected covariates were used that represent different aspects of the soil environment (Supplementary Table S1). Climatic water content (CWC) represents the soil water storage capacity and climatic water balance based on the number of consecutive dry days (DRY) and potential evapotranspiration (PET) (Supplementary Methods). It is a proxy for the soils wetness, its dynamics and aqueous phase connectivity. Both shape the number of distinct aqueous habitats and their connectedness in a soil. We found an optimal CWC in the range of 0.15 to 0.20 where bacterial richness peaks (Fig. 2a). A generally linear drop in richness seen towards low water availability is potentially due to nutrient limitations by the physically constrained diffusion processes and reduced carbon input. Soil pH exhibited a trend similar to the CWC with a peak near neutral values (pH 7, Fig. 2b) as reported in previous studies^7,21^. We note, however, a strong linear association between pH and climatic water content (*R*^2^ = 61%, *n* = 320, Fig. 2c). Climatically humid regions tend to be acidic and dry regions basic. Such trends have been attributed to the difference between mean annual precipitation (MAP) and PET that determine the climatic soil water balance for the region^24^. A net accumulation of salt in soil (e.g. in arid regions) directly results from a negative water balance with more evaporation than precipitation. This increase of mineral concentrations enhances the soil pH buffering capacity and can result in high soil pH. With an increase in ionic strength we would also expect effects on bacterial physiology (e.g. increased osmotic pressures^11,12^) and possible, specialized adaptations to these environments. A recent study attempted to disentangle the effect of salts and soil pH on bacterial community composition and revealed a strong effect of salinity^25^. This may also suggest that previously reported dependencies of bacterial diversity on soil pH^7,8,17^ could have been mediated by climatic soil water conditions via the accumulation of salts. Although pH is related to the suitability of bacterial habitats by increasing the tolerance (and competitive ability) of pH-adapted species^25^, it might not be the underlying driver of bacterial diversity. This reasoning is based on the idea that competitive exclusion can only occur with some degree of habitat overlap and interactions between species. Under most conditions in natural soils the aqueous phase is largely fragmented and the (micro-) environments experienced by bacteria are not necessarily the same. This fragmentation permits coexistence and suppresses the elimination of inferior competitors and, hence, promotes bacterial diversity. The distinct optimality of bacterial richness related to soil wetness could be attributed to (i) resource limitation for extremely dry soils and (ii) the increased habitat connectivity that suppresses diversity by promoting competitive exclusion in wet soils. In this context, pH represents the chemical niche environment, a variable under control of primary (physical) factors, i.e. resulting from a soil’s climatic water balance^24^. Temperature is another primary variable that might confound many processes. The mean annual temperature (MAT) is expected to alter species richness according to the metabolic theory of ecology^20,26^. This trend was manifested by a slight increase of richness with MAT peaking at 0–10 °C and 20–30 °C (Supplementary Fig. S1), in agreement with a previous study^4^. One explanation for the lack of clear patterns could be that temperature not only modifies growth rates of bacterial cells, but also affects habitat connectedness via effects on precipitation and water balance. This may counteract the enhancing effect of temperature on richness in wet and warm regions (e.g. the Tropics) where bacterial habitats are frequently connected. Furthermore, despite the strong variation of MAT near the soil surface, the effective range at the sampled depth of 10 cm might be narrower due to the damping effects of soil and leads to a limited range of conditions experienced in bacterial habitats. Additionally, bacteria could be able to tolerate a wide range of temperatures. Bacterial richness was found to be driven by temperature near geothermal springs only beyond 70 °C^27^; conditions that are not frequently found in soil. Nonetheless, changes in light intensity (solar radiation, RAD) are strongly correlated with temperature and latitude. A direct effect of light on bacterial richness would be expected by enabling growth of photoautotrophs and possible adaptation to high doses of UV light (or the lack thereof). Both effects could be masked by the presence of vegetation (e.g. NPP) that would intercept the solar radiation. We thus do not expect strong changes in the distribution of bacterial richness caused by light in vegetated environments and in sub surface soils (due to the strong attenuation of light). Nevertheless, the indirect effects of solar radiation should be well described by the used covariates (e.g. MAT and CWC) as light and water availability both shape the vegetation of an ecosystem. We used net primary productivity (NPP) to represent vegetation patterns at the ecosystem level and to characterize carbon input into subsurface bacterial habitats. NPP did not display a notable effect on species richness (slightly increasing richness up to 500 g C m^−2^ yr^−1^, constant richness beyond, Supplementary Fig. S1). Only in extreme environments, such as deserts and tundra, NPP seems to influence species richness.

### Multivariate general additive model (GAM) of bacterial richness

The complexity of interactions among environmental factors, vegetation and soil microorganisms suggests that a single variable alone is not likely to explain the observed patterns of soil bacterial species richness. We therefore tested the robustness of the observed single-variable trends using a multivariate general additive model (GAM) with forward selection of covariates (Table 1, Supplementary Fig. S2). The ranking of the most influential covariates remained consistent with the results of univariate GAM, with CWC slightly outperforming pH. Interestingly MAT occupied the third rank, suggesting that we were able to successfully capture combined effects on soil bacterial species richness. The goodness-of-fit statistics of the multivariate GAM using only the six selected covariates (*R*^2^ = 35%, *RMSE* = 283.7) were better than the statistics of any univariate GAM, suggesting that soil and climatic covariates provide additional information on species richness. Although we observed significant associations between bacterial diversity and environmental factors in uni- and multivariate modeling, these associations do not necessarily imply causation.

**Table 1:** Ranking of covariates determined by forward selection for the multivariate general additive model (GAM).

Step	Selected	*ΔAIC* ^a^	*p*-value^b^
1	Climatic water content	−104.64	<0.0005
2	Soil pH	−19.43	<0.0005
3	Mean annual temperature	−16.82	<0.0005
4	Silt fraction	−7.18	0.0083
5	Consecutive dry days	−1.59	0.0385
6	Cation exchange capacity	−0.08	0.1497

^a^Change of Akaike information criterion (*ΔAIC*) when the variable was added to a model that already contained the covariates listed above the current step.

^b^Likelihood ratio test of nested models.

To mitigate limitations of commonly used structural equation models (SEM) in discerning causal nonlinear effects, we have used a causal additive model (CAM)^28^ to explore potential causes of soil bacterial diversity. We used this novel approach to generate a graph of inferred structural dependencies between covariates and bacterial richness (Supplementary Fig. S3). By removing links between variables that are not considered significant (*p* ≤ 0.0005), we can distinguish direct from indirect relations between covariates and bacterial richness; as variables that remain linked to richness directly and variables that are connected to richness via others. Compared to the results of the multivariate GAM, we obtained a similar set of covariates with direct effect on species richness, i.e. CWC and DRY. Surprisingly, pH and MAT were not selected as potential direct causes, implying that they may have weaker effects on species richness or their associations with species richness were attributed to confounding effects. This approach enables further exploration of potential model structure. Nevertheless, care should be taken when interpreting inferred causal relationships as the method relies on the strong assumption of “no hidden variables” that are unknown in most natural environmental systems. Yet, it is noteworthy that no prior expectations or knowledge is imposed on the model structure, as is necessary with many SEM^17^. All direct and indirect links are deduced only from the observations with a given set of covariates. A drawback of this approach is that not all dependencies might be physically meaningful.

### Varying proportions of low abundance species

**Figure 3:**
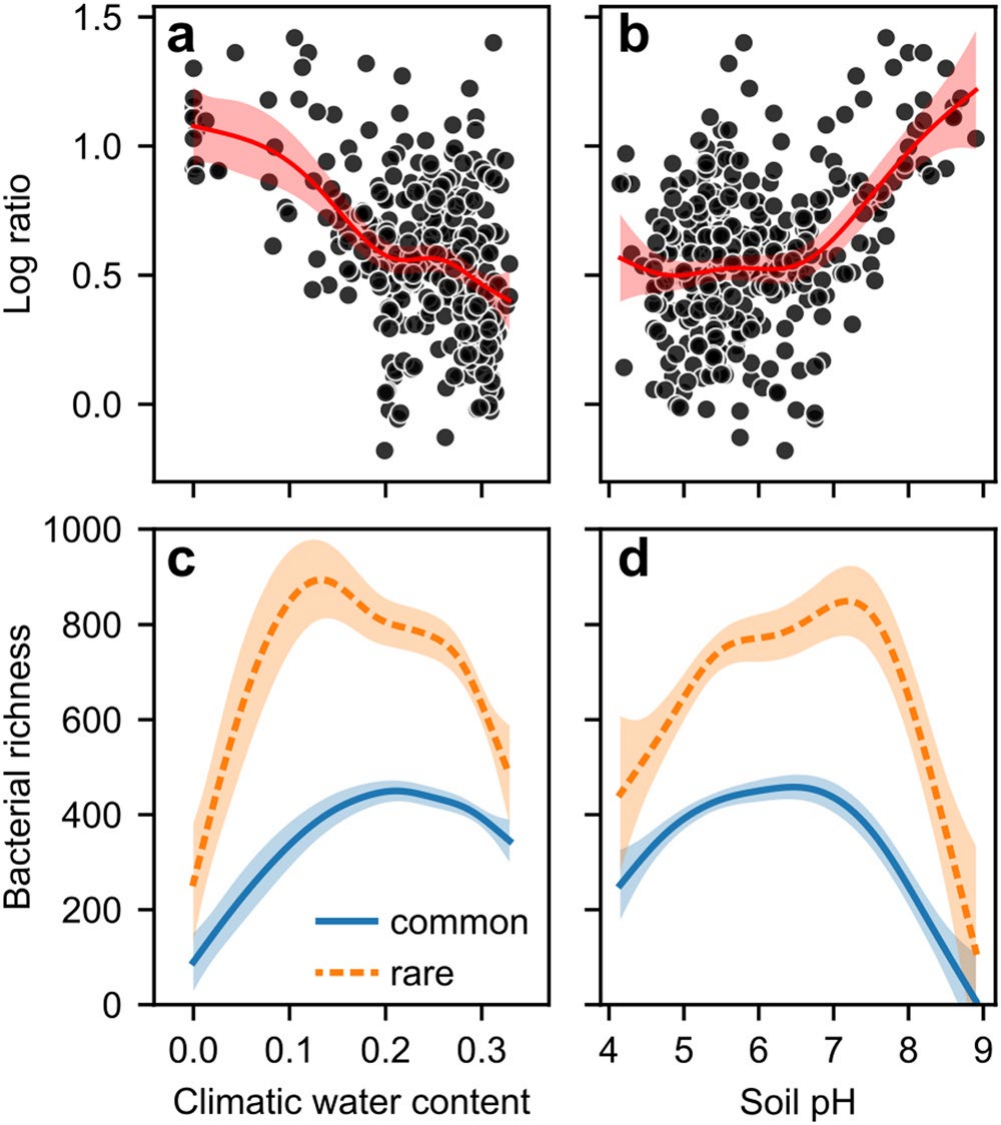
Dependence of the log-ratio of number of rare and common species per sample on the two main predictors of bacterial richness; (**a**) climatic water content (adjusted R^2^ = 24.5%, deviance explained 25.5%, AIC = 70.5, EDF = 4.3) and (**b**) soil pH (R^2^ = 23.0%, deviance explained 23.9%, AIC = 77.0, EDF = 4.1). The log ratio is calculated by splitting species into two groups based on a threshold of global relative abundance (0.005%). A log ratio of zero indicates a balanced population, where the number of rare species per sample equals the number of common species. The modeled curves of both groups richness (rare and common) are shown for (**c**) climatic water content and (**d**) soil pH. Shaded areas correspond to standard errors (n = 320).

Thus far, we have focused on explaining bacterial species richness without considering environmental effects on species with different levels of abundance. We evaluated the performance of the univariate (CWC, pH) and multivariate GAM for metrics of diversity other than species richness and found a consistent increase in *R*^2^ with increasing weight of species with low abundances (Supplementary Fig. S4). The observation indicates that species with low abundance show greater sensitivity to environmental conditions than the species dominating within samples. To further evaluate effects of environmental variables on rare and common fractions of the soil bacterial populations, we split the species in two groups by using a threshold (0.005%) of global relative abundance. For each sample, we computed the log-ratio of the number of rare and common species. A value of zero indicates that a sample contains the same number of rare and abundant species, and larger values indicate that the rare species are more numerous. We explored the dependence of the log-ratio on environmental covariates by univariate GAM (Supplementary Table S2). Interestingly we find similar, but complementary trends for CWC and pH (Fig. 3a,b). Most notably, a distinct drop in the number of rare species appears under elevated climatic soil water contents. This trend compares well with univariate and multivariate model results for species richness. The modeled dependencies of rare and common species diversity on climatic water content (Fig. 3c) demonstrate a higher susceptibility of rare species to increased aqueous phase connectivity associated with high water contents. While the common species remain abundant, the number of rare members of the soil bacterial community shows a steep decline towards wetter soil conditions. This discrepancy is weaker for soil pH where diversity of both rare and common species decrease at similar rates when approaching acidic conditions (Fig. 3d). The gradual increase in the proportion of globally rare species under drier conditions (low CWC) is likely due to the more fragmented aqueous phase that may shelter bacterial species in small but numerous isolated aqueous habitats^13,14^. Alternatively, one might argue that the emergence of rare species under basic (high pH) — and possibly also very dry— conditions is attributed to the presence of specialist phylotypes capable of coping with such an environment^8,29^. However, if neutral pH would be favored by most bacterial species (i.e. leading to more diversity) we would expect less balanced soil bacterial communities with more of the rare species present around pH 7. Interestingly, the log-ratio does not increase again towards acidic conditions. Hence, acidic environments reduce diversity of rare and common species to a similar extent, and rare (specialist) species that benefit from weaker competition with common species seem to be missing. Although, information on many additional factors that could affect the presence of rare and common species (e.g. nutrient status of the soil) could not be included in the analysis, general tendencies could be identified using the variables considered. We thus conclude that aqueous habitat connectivity largely dominates the soil bacterial richness picture and should be taken into account together with additional factors when data is available.

### Global patterns of soil bacterial richness

**Figure 4:**
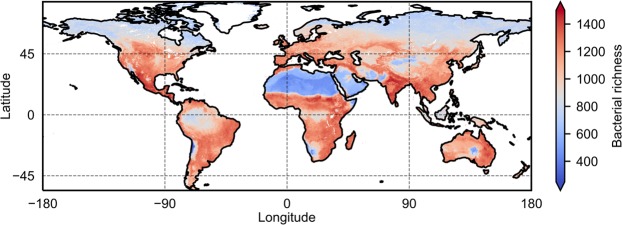
Global prediction of bacterial richness delineating spatial patterns of contrasting diversity (R^2^ = 40%, RMSE = 261.5). Tropics (e.g. Amazon, Congo) and northern higher latitudes (e.g. Siberia) show low bacterial richness. This is potentially linked to increased prevalence of frequently wet soils fostering connectedness of bacterial habitats. Low bacterial richness in deserts (e.g. Sahara, Atacama) is likely due to resource limitation. The highest bacterial richness is found in temperate regions and climatic transition zones (e.g. Sahel).

The GAM used in this study accounts only for independent and additive effects of covariates on species richness. This may not be a realistic depiction of processes in natural ecosystems with numerous connections and interdependencies. Tree-based statistical models seem better suited to account for (higher-order) interactions between variables. For prediction of global maps of species richness we therefore combined independently trained random forest and gradient boosting trees by simple averaging. The procedure was reinitialized and repeated ten times to stabilize the results and increase reproducibility. The tree-based model (*R*^2^ = 40%, *RMSE* = 261.5) performed better than the multivariate GAM (*R*^2^ = 35%, *RMSE* = 283.0) indicating that interactions between covariates are important for predicting species richness. Despite the considerably better performance, a large portion of variance remained unexplained. This is not unexpected, given the different sampling strategies and methodology of the studies. Additionally, covariates derived from remote sensing products and digital soil maps smooth the actual spatial variation of the respective characteristics and do not (yet) capture the full heterogeneity of natural soils. Another limitation of this study is the lack of fungal data. The data used does not permit analysis of fungal richness, and we can only speculate about potential, general trends. However, one study used in our dataset (EMBL)^17^ investigated fungal diversity across biomes and report that fungal diversity does not peak in temperate regions (unlike bacterial diversity). The authors further suggest niche differentiation lead to contrasting responses of fungal diversity with precipitation and soil pH compared to bacterial diversity^17^. We thus would expect fungi to play a dominant role in vegetated soils with lower pH and high C:N ratios^17,30^. Such regions (biomes) are represented by high NPP and high climatic water contents. In these environments the aqueous phase connectedness could additionally enhance competition; potentially also between bacteria and fungi. The global map of predicted bacterial richness shows distinct regions of varying bacterial richness (Fig. 4). Tropical regions (e.g. the Amazon and the Congo Basin rainforests) exhibit remarkably lower bacterial richness highlighting the adverse effects of high levels of soil wetness on bacterial diversity. Lowest richness values were also found in regions where resources are most limiting, such as in the Sahara or the Atacama deserts. “Hotspots” of species richness lie in temperate regions and climatic transition zones where resource availability is not limiting and the aqueous phase remains fragmented, such as in the northern regions of India or in the Sahel. Tree-based methods provide a complementary approach to GAM as they efficiently handle higher order interactions between covariates and provide an efficient interface for spatial mapping. The implicit representation of covariate dependencies and model averaging, however, do not offer as much insight into the model structure as is possible with GAMs.

## Conclusions

Incorporating the effects of soil and climate in the analysis of bacterial biogeography based on global datasets provides new insights into the key factors, namely climatic water content and pH that shape soil bacterial richness and community structure. The dominant role of climatic soil water content has not been fully recognized in previous studies. The inherent links between climatic soil water content and soil pH suggest that part of the soil bacterial diversity previously attributed to soil pH may reflect effects of climatic water content. We find that regions of intermediate climatic soil water content exhibit a peak in bacterial richness owing to the fragmentation of aqueous bacterial habitats that remain sufficiently supplied with resources, thus ensuring growth and protection from competitive exclusion. The results suggest that soil pH acts as a secondary driver of soil bacterial richness and represents a proxy of soil properties and climatic conditions. Placing local bacterial relative abundance in a global context provides fruitful insights into the biogeography of soil bacteria and the factors shaping spatial patterns of bacterial diversity. Especially the rare component of the soil bacterial community that contributes a large fraction of diversity is surprisingly predictable. This highlights the importance of environmental drivers, such as climatic water content, in shaping the genetic pool of potential functional capabilities by changing the size of the soil bacterial “seedbank”.

## Materials and Methods

### Data collection and processing

All 16S rRNA sequences of soil samples were obtained from three different studies. We hereafter use the terms EMBL (European Molecular Biology Laboratory)^17^, EMP (Earth Microbiome Project)^21^, and ZHOU^20^ to refer to the sources of samples and metadata. Since sequences were different in terms of their representativity and amplification protocols, filters based on sample metadata, primer sequences as well as assigned taxonomy were applied to minimize methodological differences and maximize compatibility.

### Metadata-based filtering

The metadata of soil samples (*n* = 235, 7974 and 126 for EMBL, EMP and ZHOU, respectively) were obtained from QIITA^31^ and the European Nucleotide Archive ENA^32^. Although most soil samples were initially collected with the aim to study soil microbial communities, some of them could not be considered natural. The following procedures were applied to each study:

EMP: We selected representative samples carefully based on the metadata by removing potential artificial soils (e.g. sand filter in water purification system), managed soils (e.g. agricultural soil) and soils which cannot be considered as “natural” (e.g. soil samples taken from urban environments). Further, samples of Antarctic soils and from depth >0.1 m were excluded due to limited information on local environments. The 16S rRNA sequences of all selected samples (n = 587) were retrieved from ENA.

EMBL & ZHOU: No metadata-based filtering was done since all samples could be considered representative according to the criteria applied to EMP. 16S rRNA sequences for EMBL and ZHOU were obtained from ENA using study accession ID PRJEB19856 and PRJNA308872, respectively.

### Primer-based filtering

EMBL, EMP and ZHOU used the marker gene sequencing method for amplification^33^, yet their chosen primer sets and targeted regions of 16S rRNA differed substantially. To avoid primer biases, we only included samples which amplified the V4 region of 16S rRNA. Furthermore, two slightly different primer sets were used between studies, i.e. the original 515F-806R primer^34^ and its modification^35^. The original primer (forward: GTGCCAGCMGCCGCGGTAA, reverse: GGACTACHVGGGTWTCTAAT) is known to be biased towards certain archaeal and bacterial groups, such as Crenarchaeota, Thaumarchaeota and SAR11^36,37^. The modified one adds one degeneracy in both the forward (GTGYCAGCMGCCGCGGTAA) and reverse (GGACTACNVGGGTWTCTAAT) primer to reduce those biases. However, most samples in EMP and ZHOU were published before the modified primer set came in use, whereas all samples in EMBL were amplified using the modified one. To make a valid comparison, we either filtered particular sequences which could only be captured by the modified primer set (if the primers were retained in the raw sequences), or dropped the entire sample (if no information was available about the primers). We additionally removed sequences in which adapters could be identified (adapter contamination).

#### EMBL

All sequences in EMBL were raw and unjoined. We discarded pairs of sequences if GTGTCAGCMGCCGCGGTAA could be found in the forward reads or GGACTACGVGGGTWTCTAAT in the reverse reads (difference between the original and the modified primer). The forward and reverse reads were subsequently joined, trimmed and quality controlled (Phred threshold of three) using VSEARCH (QIIME2, 2018.8.0)^38^, cutadapt^39^ and split_libraries_fastq.py (QIIME1, 1.9.1)^34^, respectively.

#### EMP and ZHOU

Unlike EMBL, sequences in EMP and ZHOU obtained from ENA were already preprocessed, i.e. de-multiplexed, and trimmed. Both of them were quality filtered with a Phred threshold of three using the script split_libraries_fastq.py (QIIME1, 1.9.1)^34,40^.

### Denoising

The Deblur (1.1.0) algorithm^41^ was chosen to de-replicate sequences and remove potential sequencing errors. All sequences were trimmed to a length of 90 base pairs since most sequences in EMP had a length of 90 bases pairs, and the algorithm requires all sequences to have the same length. To strengthen the filtering rules, singletons per sample were removed before denoising by setting the min_size parameter to two. The algorithm corrected sequences based on a predefined error profile and returned amplicon sequence variants (ASV), which could be considered as putative error-free (representative) sequences for each sample. We adopted a method based on ASV instead of clustering sequences into operational taxonomic units (OTUs) because ASVs are (i) consistently labeled, thus facilitating meta-analysis of cross-study samples, and (ii) are not affected by the incompleteness of reference databases, hereby providing more accurate diversity estimates for bacterial communities^42–44^. A total of 256,620 unique ASV were identified with most of the sequences being relatively rare (14.94% observed only once and 70.79% less than ten times across all soil samples).

### Taxonomy assignment for filtering of archaea

ASVs were assigned to taxonomic units using a multinomial Naive Bayes classifier (QIIME2, 2018.8.0), trained on the Greengenes 13_8^45^, 99% OTUs (515F-806R region, 90 base pairs). Nevertheless, only 1.08% of the sequences could be assigned to a unique species designation. Sequences which were classified as archaea were removed, as they only contributed to a small proportion and may behave differently from bacteria^46^. Sequences that could not be classified confidently (<70%) at the lowest taxonomic levels (Kingdom) were discarded. Global singletons (observed only once across all samples) were dropped to remove potential errors and increase reliability.

### Rarefaction and estimation of diversity

The optimal sequence rarefication depth (number of randomly drawn sequences without replacement from each sample) with respect to diversity was determined by a grid search over 2,500 to 15,000 (Supplementary Fig. S5). After determining the rarefication depth, the procedure was repeated 100 times to increase reproducibility of ASVs abundance distributions. For each soil sample, diversity indices were calculated independently for each of the 100 rarefied ASVs tables and subsequently averaged^17^. The abundance of each ASVs was averaged over the 100 rarefied species abundance distributions, and thus may not be integer valued. We note that this procedure differs from common practices in ecological fields in which only one randomly generated rarefied ASVs (or OTUs) table is used for both diversity estimation and interpretation. From an ecological point of view, the randomness in the latter approach can be desired since in reality we would not have the ability to take multiple soil samples from the same site, amplify them independently and take the averaged diversity (corresponding to rarefying multiple times from an existing ASV or OTU table). However, from a statistical point of view, it lacks stability. In the foregoing analysis, we used the averaged (n = 100) 7,500 ASVs as representative phylotypes for calculations of bacterial diversity in its general form (Supplementary Methods).

### Covariates

Soil properties were collected from 250 m SoilGrids^47^ according to samples’ geographical locations and soil depth. We did not use the on-site measured soil properties due to missing values and inconsistent methodologies of measurement across studies. Of additional concern was the comparison of variables measured at different scales. While it is common practice to compare remotely sensed covariates (e.g. temperature, primary productivity, precipitation) with sample scale measurements (e.g. pH, carbon-, nitrogen content) it is not desirable from a statistical point as the level of support varies. This can lead to misinterpretation of the relative variable importance with respect to their explanatory power and hereby would obscure our understanding of underlying processes. The mean annual net primary (NPP) productivity, obtained from MODIS 2000–2015^48^ was used as a proxy for the net carbon influx and the distribution of land covers. Mean annual temperature (MAT) and solar radiation (RAD) were retrieved from WorldClim^49^. Mean annual precipitation (MAP) was estimated using MSWEP rainfall data^50^. Using mean monthly temperature and shortwave radiation as inputs, mean monthly potential evapotranspiration (PET) was calculated according to the empirical equation proposed by Jensen and Haise^51^. The empirical equation produced negative values at extremely low temperatures. These estimated negative PET are unrealistic and were replaced by zeros. The resulting mean monthly PET was averaged over one climatic year yielding the mean annual PET. The average number of consecutive dry days (DRY) was estimated from the MSWEP precipitation time series. Briefly, daily precipitation was compared against the mean annual potential evapotranspiration (PET) to detect rainfall events that were expected to alter soil moisture conditions, i.e. exceeding the threshold set by PET. The values were reported as an absolute averaged spacing between rainfall events and could exceed one year. The available water capacity (AWC) in SoilGrids was derived based on a pedo-transfer function that depends on soil chemical conditions, e.g. soil pH (PH)^47^. Including soil chemistry in calculating AWC may potentially interfere with later interpretations. To avoid this, we alternatively estimated AWC by a function that only uses bulk density (BLD), organic carbon content (ORC), silt content (SLT) and clay content (CLY)^52^. Climatic water content (CWC) was introduced to describe the climatic state of soil wetness (Supplementary Methods). It was calculated based on the assumption that the top one meter of soil can be fully replenished up to field capacity during rainfall events, and dry exponentially in consecutive days without rain (DRY). Summary of covariates is given in the Supplementary Table S1.

### Correlation and clustering

Spearman’s rank correlation *ρ*_*s*_ was used to measure the pairwise correlation between covariates (Supplementary Fig. S6). Covariates were then hierarchically clustered^53^ according to their dissimilarity (distance), defined as $1-|{\rho }_{s}|$. The inter-cluster distance was determined by the averaged dissimilarity of objects in different clusters (average linkage). The cluster size was selected by applying a dissimilarity threshold of 0.15. Within each cluster, only one covariate with the simpler physical interpretation was retained (Supplementary Fig. S6). Further, since sand (SND), silt (SLT) and clay content (CLY) are compositional, SND was discarded.

### Generalized additive models

Generalized additive models (GAM) (R package mgcv, 1.8–24) were used to model the associations in both univariate and multivariate analysis^54^. Thin plate regression spline was chosen as basis function and the smoothing parameters were estimated by restricted maximum likelihood (REML). The dimension of the basis used for each smoothing term was not restricted (default parameter *k*). Forward selection in multivariate modeling was performed based on Akaike information criterion (*AIC*) and likelihood ratio tests (conditional on the estimated smoothing parameters). The double penalty approach of GAM was used for regularization. Covariates were considered as negligible in terms of contributions to model fits if their estimated degree of freedom were shrunk approximately to zero (<10^−3^). The prediction performance was evaluated using leave-one-out cross-validated coefficient of determination (*R*^2^) and root mean squared error (*RMSE*).

### Causal additive models

Causal additive models (CAM) (R package CAM, 1.0) were used to infer the underlying data generating mechanism (causal structure) from observational data^28^. The model is a special case of the general structural equation model (SEM)^55^, namely in that the structural equations are additive in variables and errors. The model further assumes no hidden variables, i.e. all variables involved in the data generating mechanism are observed, and absence of directed cycles in the causal graph. Since the dimension of the dataset was low (15 covariates, except SND), we did not use preliminary neighborhood selection (screening of covariates primarily aimed for reduction of computational time). Furthermore, in order to avoid using data twice (for both variable selection and inference after selection)^56,57^, as well as the issue of “*p*-value lottery”^58–60^, the last step (pruning of the directed graph) of CAM was combined with the multi-splits method^59^. Briefly, the method randomly splits data into training and testing sets; the training set is used for estimating the graph structure while the testing set is used for computing *p*-values of each covariate (repeated 100 times to avoid noisy selection of covariates and to stabilize the results).

### Prediction of global maps using tree-based algorithms

Random forests (RF) (RandomForestRegressor in scikit-learn, 0.19.1) and gradient boosting trees (GB) (GradientBoostingRegressor in scikit-learn, 0.19.1) were used for prediction^61^. Hyperparameters (n_estimators, max_features, max_depth and min_samples_leaf) in both algorithms were optimized using cross validation (CV) with respect to *R*^2^. Additionally, the learning rate in the boosting algorithm was set to a constant value of 0.05 since it can be compensated by the number of iterations. Independently trained random forest and gradient boosting trees were stacked by simple averaging. The generalization errors (*R*^2^ and *RMSE*) were estimated using nested (ten by ten folds) CV, i.e. the inner CV selected the best-fit models (optimizing hyperparameters with respect to *R*^2^) while the outer CV computed the test errors of the selected models. The entire procedure was repeated ten times using different random splits (or seeds) to increase stability. Using the estimated model we predicted global bacterial richness at the full spatial coverage of covariates.

## Supplementary information

Supplementary Information

## Data Availability

All generated data and code used for the analysis are available from the corresponding author upon request. Computer code necessary to reproduce the main findings is accessible online (https://gitlab.ethz.ch/bickels/biogeo) and is archived in a public repository with DOI 10.5281/zenodo.3366252.
