# Supplementary material for: A hierarchy of environmental covariates control the global biogeography of soil bacterial richness
Source: Sci Rep. 2019 Aug 20;9:12129. doi: 10.1038/s41598-019-48571-w (PMC6702155; doi:10.1038/s41598-019-48571-w)
Supplement: Supplementary file 1 — Supplementary Information [file 41598_2019_48571_MOESM1_ESM.pdf]

Supplementary Information (SI):

A hierarchy of environmental covariates control the global biogeography of soil bacterial richness

Authors

- Samuel Bickel[1,*] (samuel.bickel@usys.ethz.ch)
- Xi Chen[1] (xiche@student.ethz.ch)
- Andreas Papritz[1] (andreas.papritz@env.ethz.ch)
- Dani Or[1] (dani.or@env.ethz.ch)

Author Affiliation

[1]Soil, Terrestrial and Environmental Physics (STEP); Institute of Biogeochemistry and Pollutant dynamics (IBP); Swiss Federal Institute of Technology (ETH), 8092 Zürich

[*]Corresponding author: SB (samuel.bickel@usys.ethz.ch)

SI Figures

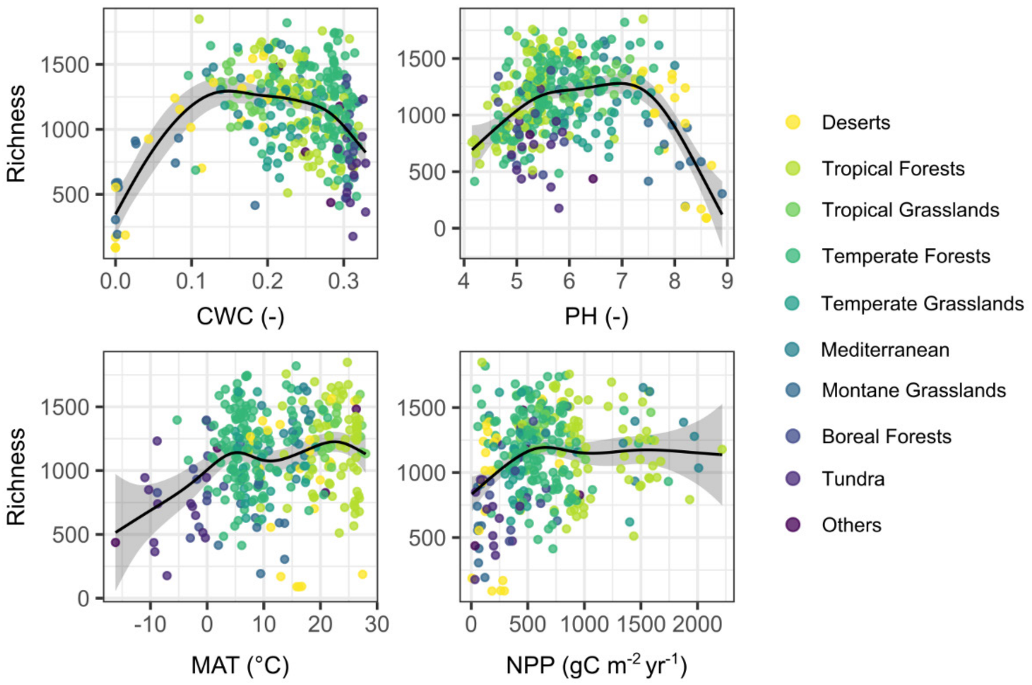

SI Fig. S1: Univariate GAM of selected variables. Colors indicate the sampled biomes. Bacterial richness as a function of climatic water content (CWC; R^2 = 27.7%, *RMSE* = 298.1, *AIC* = 4557.5, *EDF* = 4.7), soil pH (PH; R^2 = 23.8%, *RMSE* = 306.0, *AIC* = 4574.0, *EDF* = 5.1), mean annual temperature (MAT; R^2 = 5.9%, *RMSE* = 340.0, *AIC* = 4640.6, *EDF* = 4.9) and net primary productivity (NPP, R^2 = 5.7%, *RMSE* = 340.5, *AIC* = 4642.4, *EDF* = 3.7). Colors indicate the sampled biomes. Shaded areas correspond to standard errors (n = 320).

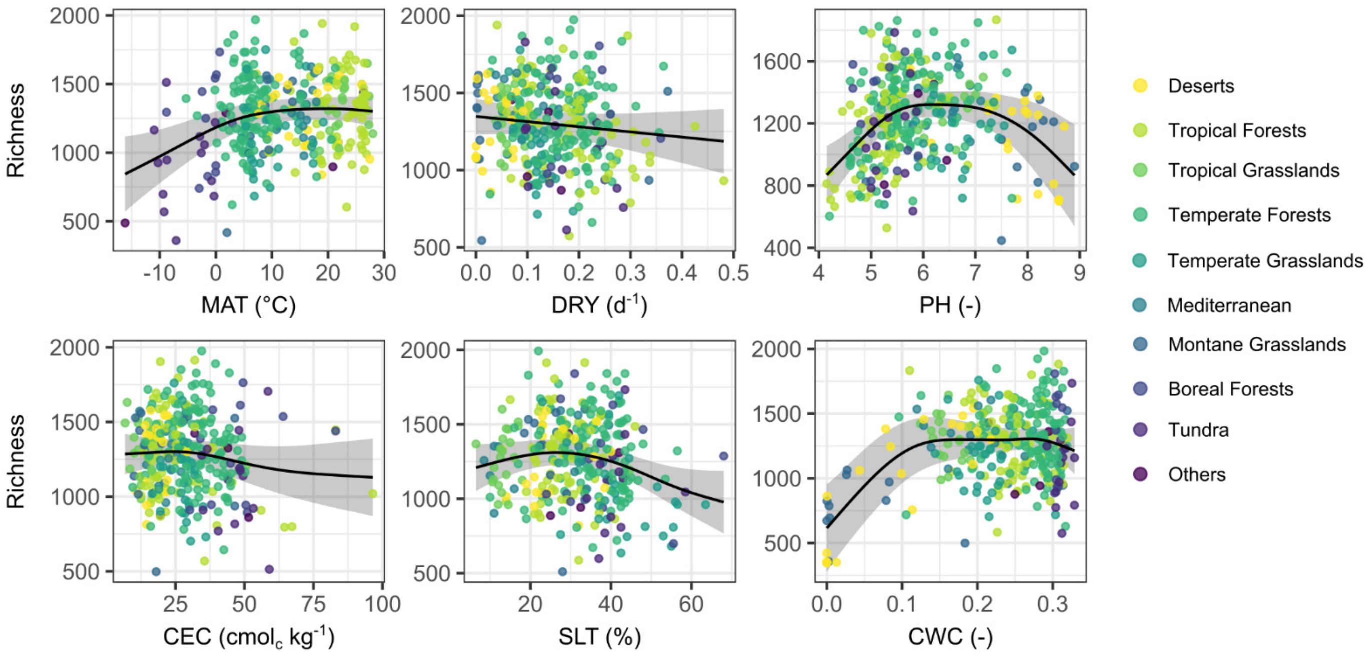

SI Fig. S2: Partial dependence plots of multivariate GAM for covariates temperature (MAT), frequency of dry days (DRY), soil pH (PH), cation exchange capacity (CEC), silt content (SLT), and climatic water content (CWC). Colors indicate the sampled biomes. Shaded areas correspond to standard errors (R^2 = 34.5%, *RMSE* = 283.6, *AIC* = 4517.8, n = 320).

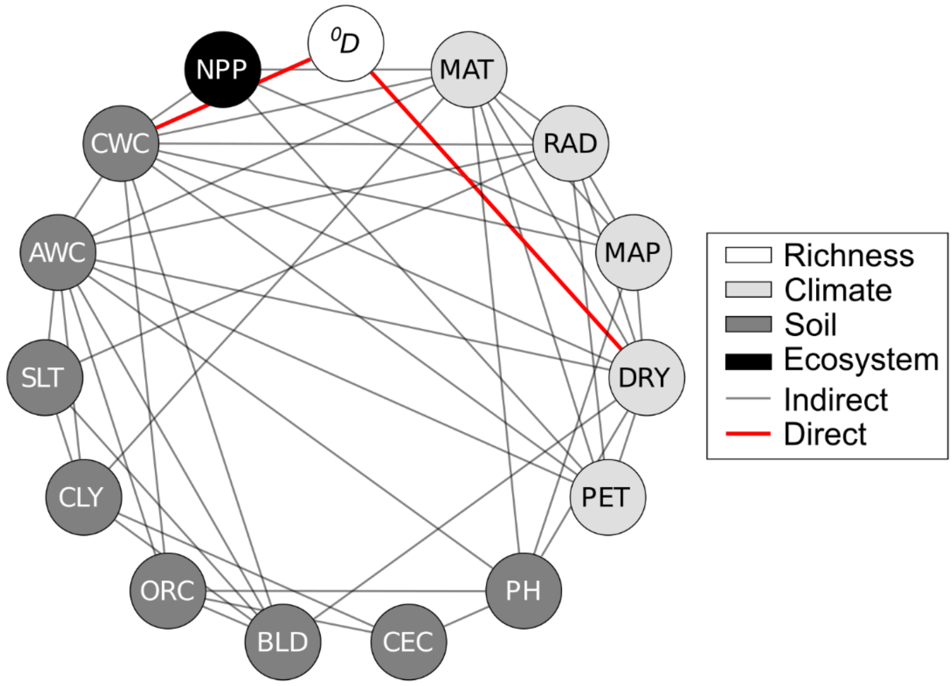

SI Fig. S3: Graph of dependencies estimated by the causal additive model (CAM) algorithm. Covariates are grouped by climate, soil and ecosystem properties. Bacterial richness (^{0}D) is the variable of interest and edges indicate inferred causal dependencies ($p \leq 0.0005$). The direct edges to soil bacterial richness are shown in red while all indirect edges are shown in grey.

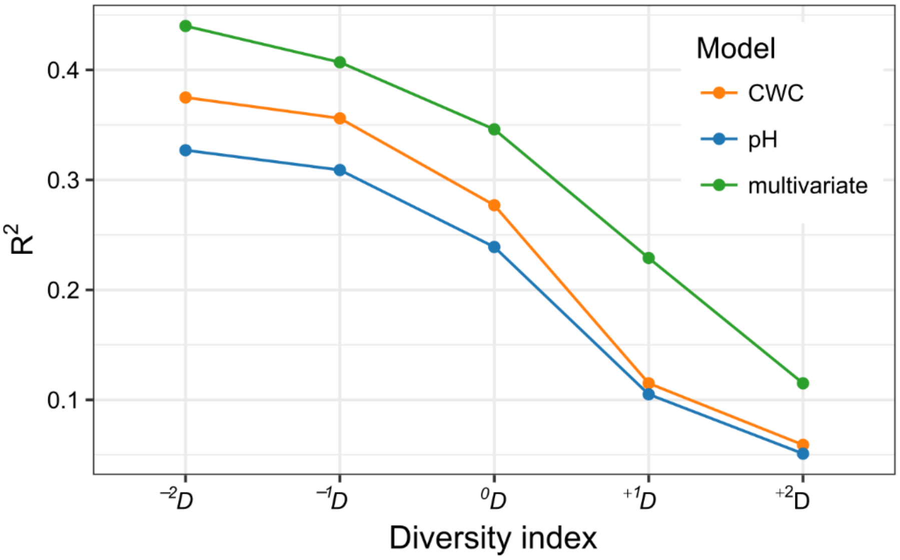

SI Fig. S4: Change of goodness-of-fit (*R^2*) of univariate (climatic water content CWC, pH) and multivariate GAM for diversity indices ^{q}D that give dominant species more weight by increasing order *q* of the index.

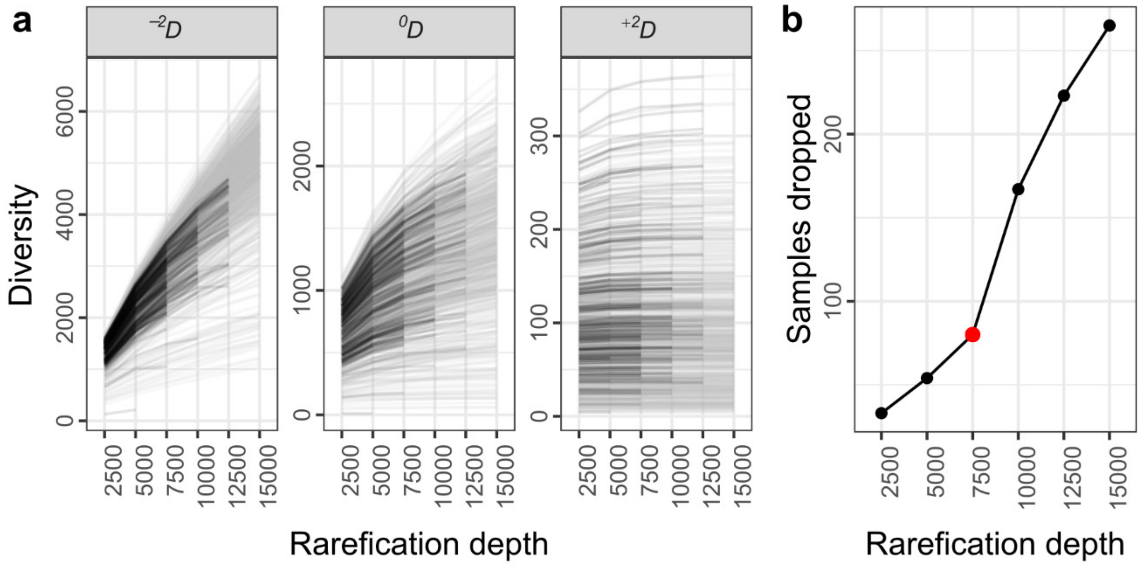

SI Fig. S5: Species accumulation curves for varying rarefication (sampling) depths. (**a**) Different diversity metrics show varying response to sampling depth. More weights on abundant species (^{+2}D) leads to saturation of the metric with smaller rarefication depth. (**b**) Choice of sampling depth (red point) as a trade-off between the numbers of dropped samples and maximized rarefication depth.

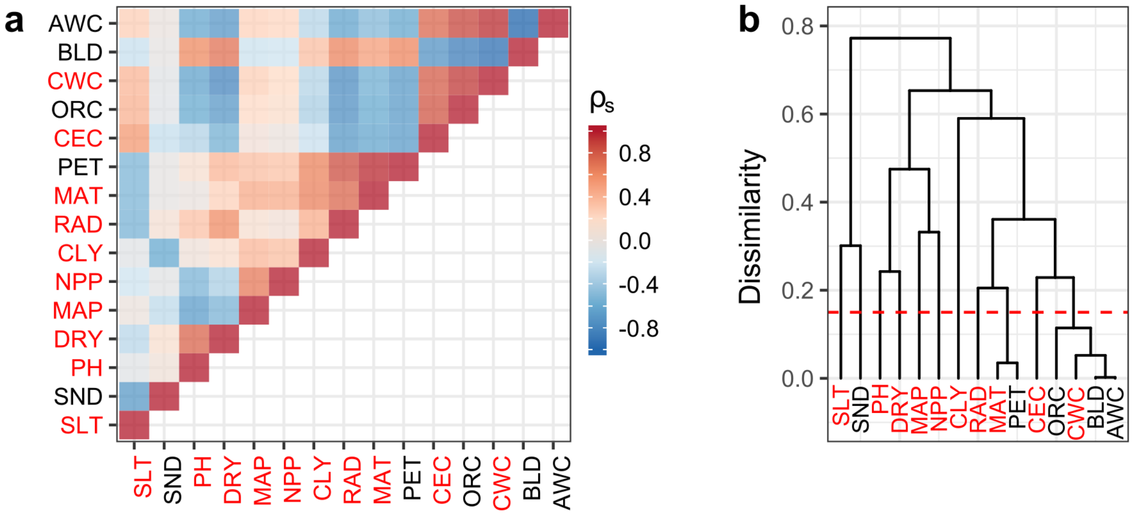

SI Fig. S6: Spearman correlation among covariates. (**a**) Matrix of pairwise correlation coefficients. (**b**) Hierarchical clustering of covariates based on their dissimilarity. From insufficiently dissimilar covariates (<0.15) only a single covariate (red) was chosen for modelling.

SI Tables

SI Table S1: Summary of covariates and their input data.

Covariate	Unit	Description	Input
MAT	°C	Temperature	WorldClim v2 [1]
RAD	kJ m^{-2} d^{-1}	Solar radiation	WorldClim v2 [1]
MAP	mm yr^{-1}	Precipitation	MSWEP v2.2 [2]
DRY	d	Number of consecutive dry days	PET, MSWEP v2.2 [2]
PET	mm d^{-1}	Potential evapotranspiration	*f*(MAT, RAD) [3]
PH	-	Soil pH	SoilGrids [4]
CEC	$cmol_c$ kg^{-1}	Cation exchange capacity	SoilGrids [4]
BLD	kg m^{-3}	Bulk density	SoilGrids [4]
ORC	g kg^{-1}	Organic carbon content	SoilGrids [4]
CLY	%	Clay content	SoilGrids [4]
SLT	%	Silt content	SoilGrids [4]
SND	%	Sand content	SoilGrids [4]
AWC	-	Available water-holding capacity	*f*(BLD, ORC, SLT, CLY) [5]
CWC	-	Climatic water content	*f*(PET, DRY, AWC)
NPP	g C m^{-2} yr^{-1}	Mean net primary productivity (2000-2015)	MODIS17 [6]

SI Table S2: Leave one out cross-validated test errors of the log ratio of bacterial richness with different (global) relative abundance cutoffs.

	**Log ratio** := $log(N_{rare}/N_{common})$		
Global relative abundance cutoff	***0.0005%***	***0.005%***	***0.05%***
MAT	15.8%	11.4%	12.2%
RAD	18.6%	23.8%	22.9%
MAP	12.5%	16.7%	12.2%
DRY	11.3%	19.5%	19.2%
PH	10.6%	21.2%	21.5%
CEC	17.9%	16.4%	15.6%
CLY	-1.0%	-0.3%	-0.9%
SLT	15.4%	20.3%	20.4%
CWC	11.7%	22.3%	24.3%
NPP	14.1%	14.6%	6.7%

SI Methods

Calculation of climatic water content

Climatic water content (CWC), was introduced to approximately describe the state of soil wetness specific to climate and soil storage capacity. It was calculated based on the assumption that the top one meter of soil (d_{soil} = 1 m) can be fully replenished up to field capacity (θ_{FC} defined as half porosity/AWC) during rainfall events, and drain exponentially in consecutive dry days (DRY). During this time, water mass is lost at a constant rate determined by (mean daily) potential evapotranspiration (PET) resulting in an exponential reduction of average water content. The MSWEP [2] precipitation records of 37 years (1979–2016) are used at daily resolution to derive average rainfall quantities per wetting-drying cycle. The precipitation time series is subjected to a threshold taken from estimates of PET to identify wetting events. The metric used is the mean time interval between rainfall events (an ensemble average) τ. This quantity combined with daily PET (m d^{-1}) lead to the following expression for climatic water content θ_τ:

$$\theta_\tau = \theta_{FC} e^{-\alpha<\tau>} \text{ with } \alpha = \frac{PET}{d_{soil}\,\theta_{FC}}$$

Diversity indices

Diversity of ecological communities can be quantified from different aspects, e.g. richness measures the number of unique types present in a community, while evenness compares the relative abundances that make up the local community[7]. Here, to measure how diverse a local community is, we opted for Hill's diversity ${}^{q}D$[8], defined as:

$${}^{q}D = \left(\sum_{i=1}^{N} p_i^q\right)^{1/(1-q)}$$

where p_i refers to the relative abundance (with $\sum p_i = 1$) of the ith type and N is the total number of types in the population. The order q controls the weights given to species of different local abundance, i.e. fewer weights will be given to the rarities if $q > 0$, and vice versa. ${}^{0}D$ (richness) simply counts unique types in a population and thus gives equal weight to all species regardless of local abundance. ${}^{1}D$ and ${}^{2}D$ are closely related to the Shannon index[9], as a limiting case for q=1 and the Simpson index[10], respectively. Therefore, ${}^{1}D$ is less sensitive to low abundant species in local communities compared to ${}^{0}D$, while ${}^{2}D$ is the least sensitive and can be considered as a measure of dominant species[11,12]. Their counterparts, i.e. ${}^{-2}D$ and ${}^{-1}D$ were also included to get a full picture of the local abundance distribution.

References

1. Fick, S. E. & Hijmans, R. J. WorldClim 2: new 1-km spatial resolution climate surfaces for global land areas: new climate surfaces for global land areas. *Int. J. Climatol.* **37**, 4302–4315 (2017).
2. Beck, H. E. *et al.* MSWEP: 3-hourly 0.25° global gridded precipitation (1979-2015) by merging gauge, satellite, and reanalysis data. *Hydrol. Earth Syst. Sci. Discuss.* 1–38 (2016). doi:10.5194/hess-2016-236
3. Jensen, M. E. & Haise, H. R. Estimating Evapotranspiration from Solar Radiation. *Proc. Am. Soc. Civ. Eng. J. Irrig. Drain. Div.* **89**, 15–41 (1963).
4. Hengl, T. *et al.* SoilGrids250m: Global gridded soil information based on machine learning. *PloS One* **12**, e0169748 (2017).
5. Tóth, B. *et al.* New generation of hydraulic pedotransfer functions for Europe: New hydraulic pedotransfer functions for Europe. *Eur. J. Soil Sci.* **66**, 226–238 (2015).
6. Zhao, M., Heinsch, F. A., Nemani, R. R. & Running, S. W. Improvements of the MODIS terrestrial gross and net primary production global data set. *Remote Sens. Environ.* **95**, 164–176 (2005).
7. Stirling, G. & Wilsey, B. Empirical Relationships between Species Richness, Evenness, and Proportional Diversity. *Am. Nat.* **158**, 286–299 (2001).
8. Hill, M. O. Diversity and Evenness: A Unifying Notation and Its Consequences. *Ecology* **54**, 427–432 (1973).
9. Shannon, C. E. A Mathematical Theory of Communication. *Bell Syst. Tech. J.* **27**, 379–423 (1948).
10. Simpson, E. H. Measurement of Diversity. *Nature* **163**, 688 (1949).
11. Whittaker, R. H. Dominance and Diversity in Land Plant Communities. *Science* **147**, 250 (1965).
12. Locey, K. J. & Lennon, J. T. Scaling laws predict global microbial diversity. *Proc. Natl. Acad. Sci.* **113**, 5970–5975 (2016).
